# Imaging of Endometriotic Lesions Using cRGD-MN Probe in a Mouse Model of Endometriosis

**DOI:** 10.3390/nano14030319

**Published:** 2024-02-05

**Authors:** Nazanin Talebloo, M. Ariadna Ochoa Bernal, Elizabeth Kenyon, Christiane L. Mallett, Sujan Kumar Mondal, Asgerally Fazleabas, Anna Moore

**Affiliations:** 1 Precision Health Program, Michigan State University, 766 Service Road, East Lansing, MI 48824, USA; talebloo@msu.edu (N.T.); kenyonel@msu.edu (E.K.); mondalsu@msu.edu (S.K.M.); 2 Department of Chemistry, College of Natural Sciences, Michigan State University, 578 S Shaw Lane, East Lansing, MI 48824, USA; 3 Department of Obstetrics, Gynecology & Reproductive Biology, Michigan State University, 400 Monroe Avenue NW, Grand Rapids, MI 49503, USA; ochoaber@msu.edu (M.A.O.B.); fazleaba@msu.edu (A.F.); 4 Department of Animal Science, Michigan State University, 474 S Shaw Ln #1290, East Lansing, MI 48824, USA; 5 Department of Radiology, College of Human Medicine, Michigan State University, 766 Service Road, East Lansing, MI 48824, USA; cmallett@msu.edu; 6 Institute for Quantitative Health Science and Engineering, Michigan State University, 775 Woodlot Drive, East Lansing, MI 48824, USA

**Keywords:** endometriosis, lesions, integrins, nanoparticle, contrast agent, magnetic resonance imaging

## Abstract

Approximately 10% of women suffer from endometriosis during their reproductive years. This disease is a chronic debilitating condition whose etiology for lesion implantation and survival heavily relies on adhesion and angiogenic factors. Currently, there are no clinically approved agents for its detection. In this study, we evaluated cRGD-peptide-conjugated nanoparticles (RGD-Cy5.5-MN) to detect lesions using magnetic resonance imaging (MRI) in a mouse model of endometriosis. We utilized a luciferase-expressing murine suture model of endometriosis. Imaging was performed before and after 24 h following the intravenous injection of RGD-Cy5.5-MN or control nanoparticles (Cy5.5-MN). Next, we performed biodistribution of RGD-Cy5.5-MN and correlative fluorescence microscopy of lesions stained for CD34. Tissue iron content was determined using inductively coupled plasma optical emission spectrometry (ICP-OES). Our results demonstrated that targeting endometriotic lesions with RGD-Cy5.5-MN resulted in a significantly higher delta T2* upon its accumulation compared to Cy5.5-MN. ICP-OES showed significantly higher iron content in the lesions of the animals in the experimental group compared to the lesions of the animals in the control group. Histology showed colocalization of Cy5.5 signal from RGD-Cy5.5-MN with CD34 in the lesions pointing to the targeted nature of the probe. This work offers initial proof-of-concept for targeting angiogenesis in endometriosis which can be useful for potential clinical diagnostic and therapeutic approaches for treating this disease.

## 1. Introduction

Endometriosis is an inflammatory gynecological disorder which is marked by the persistent development and presence of ectopic tissue resembling endometrium outside the uterine cavity. This chronic disorder affects approximately 10% of women throughout their reproductive years and tends to show a progressive nature [1,2,3]. There is no single symptom that defines endometriosis, as it can be accompanied by a range of different symptoms including pelvic pain, heavy menstrual bleeding, dysmenorrhea, and infertility [2,4,5]. Endometriosis is a heterogeneous disease with a complex etiology [4,6] which makes it difficult to diagnose. Several theories have been proposed to elucidate the establishment and development of endometriotic lesions. However, as of today, there is no single theory that explains the variety of the different clinical presentations of endometriosis [7]. At present, the clinical gold standard for diagnosing endometriosis involves diagnostic laparoscopy with histological confirmation to validate the presence of endometriotic glands and stroma. Still, there has been a recent paradigm shift in evaluating this approach since this procedure is invasive with potential for complications. In the absence of a planned surgical intervention to remove lesions, it is now recommended to consider other non-invasive diagnostic methods and conduct a thorough physical examination along with an evaluation of the patient’s medical history [8,9]. Since the development of non-invasive and low-invasiveness methods for diagnosing endometriosis poses a challenge, the potential of other diagnostic methods—including genetic tests, biomarkers, and imaging—has been assessed [10,11,12,13,14,15]. Imaging technologies that are currently being used to help diagnose this condition are ultrasound (especially transvaginal ultrasound) and magnetic resonance imaging (MRI) [16,17]. However, ultrasound is generally operator-dependent and cannot detect all the lesions while MRI has the potential to overlook certain endometriotic masses [18,19,20,21,22,23,24]. Despite the implementation of dedicated and specialized MRI protocols which incorporate both T1 and T2-weighted sequences, this imaging modecan readily miss endometriotic lesions due to their small and abnormal signal presentation [20,21,22,23,24]. Designing specific contrast agents can aid in the MRI-based diagnosis of endometriosis by enhancing the signal contrast between lesions and adjacent tissues [25]. Several studies have investigated targeted approaches to deliver therapeutics or imaging agents to endometriotic lesions [22,26,27,28]. However, currently there are no contrast agents for endometriosis detection that have received clinical approval [22,29]. Importantly, the goal is to identify the lesions in their early stages, which is particularly challenging when using MRI trying to pinpoint the lesions with specific traits and locations. It is those early lesions that are so essential to detect since later-stage lesions show more characteristic patterns in terms of pain and presentation and are easier to detect. By focusing on the targeted approach, the signal enhancement in MR images can be improved, leading to more precise lesion detection at the earlier stage. Consequently, initiating therapeutic interventions during these earlier stages becomes possible, which minimizes symptoms and improves outcome.

Angiogenesis is a complex process which supports the development of a new vascular system which sprouts from the existing vasculature via the interaction between the cellular matrix, proteolytic enzymes, and cytokines. This cascade plays a crucial role in the progression of endometriosis due in part to the inflammation and formation of new vessels, which are necessary for delivering nutrients and an oxygen supply to the lesions [30,31]. The distinct interplay between cytokines, growth factors, and angiogenic factors aids in establishing and progressing endometriotic implants (reviewed in [32]). Interestingly, a recent study showed that cytokine secretion may promote angiogenesis in neighboring lesions via paracrine actions, which contributes to the development of endometriosis [33]. Generally, angiogenesis is regulated and mediated by integrins, which are members of a family of cell surface adhesion receptors controlling adhesive interactions of vascular cells [34,35]. Alpha(v)beta3 integrin, a receptor for both fibronectin and vitronectin, plays a major role in blood vessel formation and is significantly overexpressed on activated endothelial cells during angiogenesis compared to quiescent endothelial cells [36,37]. It plays a major role in the process of lesion attachment and is overexpressed in established endometriotic lesions. RGD peptides, which contain an arginine–glycine–aspartic acid sequence, are well known to bind preferentially to the alpha(v)beta3 and alpha5beta1integrins [38,39]. Targeting angiogenesis and cell adhesion in lesions using imaging probes may improve the reliability of detecting endometriosis. Here, we proposed using cyclic RGD (c-RGD) peptide for targeting endometriotic vasculature similarly to what has been done in cancer cases [38,40,41]. To deliver the RGD targeting moiety to the lesions we utilized superparamagnetic iron oxide nanoparticles that are known to be biocompatible, biodegradable and have been used for a wide variety of medicinal and biomedical applications such as tumors or vascular imaging, and therapeutics delivery [42,43,44]. Decorating the nanoparticle surface with various ligands, such as specific peptides, can facilitate binding to a biomarker that is selectively overrepresented in targeted cells and reduces off-target accumulation [45]. Due to the superparamagnetic nature of the iron core of these nanoparticles, they can serve as T2 contrast agents for magnetic resonance imaging (MRI), and their accumulation can be visualized as a darkening of the tissues on T2-weighted MR images [44]. Developing nanoparticle-based targeted contrast agents can help in precise diagnosis of endometriosis as well as in delivering potential therapeutics to the lesions while reducing off-target effects (theranostics). Previous studies have demonstrated a considerable increase (over 15-fold) in the blood half-life of dextran-coated iron oxide nanoparticles conjugated with RGD (similar to the probe presented in our study) as compared to the unconjugated peptide [40]. Previously, the administration of a similar conjugate in a mouse tumor model demonstrated increased accumulation of the targeted probe compared to the control probe. This observation confirms the conjugate’s stability in circulation and its successful delivery to the tumor site [46]. Similar dextran-coated iron oxide nanoparticles have been previously employed in clinical settings for identification of lymph node metastases in humans with metastatic prostate cancer [47]. We expect that because of the previous history of using these nanoparticles in clinical settings, our translation into clinics will be straightforward.

Here, we demonstrate for the first time the use of c-RGD-conjugated, iron oxide nanoparticles labeled with a near-infrared optical dye Cy5.5 (RGD-Cy5.5-MN) for imaging of endometriotic lesions in a murine model of endometriosis using MRI. Our investigation demonstrated targeted delivery of nanoparticles confirmed by histology and elemental analysis.

This study provides the initial proof of utilizing c-RGD-targeted iron oxide nanoparticles for imaging of endometriosis with magnetic resonance imaging in a model of endometriosis in which the pieces of the endometrium were sutured in place. The potential of this probe to deliver therapeutics to lesions via targeted delivery should be further investigated.

## 2. Materials and Methods

### 2.1. Mouse Model of Endometriosis

Ten-week-old female Pgr*^cre/+^* Rosa26*^Luc/Luc^* and Pgr*^cre/+^* Rosa26*^Luc/+^* mice ([48]; n = 9) underwent a 3-day treatment with 17b estradiol (E2, Sigma-Aldrich, St. Louis, MO, USA; 1 mg/mL in oil, 0.1 µg/mouse/day) to synchronize the estrus cycle and facilitate lesion growth. Following the final E2 injection, endometriosis was induced by removing one uterine horn from each mouse. This was done by making a midline abdominal incision, cutting the caudal end of the uterine horn near the uterotubal junction, and closing the lesion with sterile absorbable suture. The excised uterine horn was cut longitudinally, and pieces of tissue were acquired using a 2 mm dermal biopsy punch. Three biopsy samples were sutured to each side of peritoneal wall (total n = 6) with a 7-0 braided silk suture. The muscle layer was closed as one layer, followed by the closure of the skin as a second layer. Subsequent to the surgery, the abdominal incision was closed. Pgr*^cre/+^* Rosa26*^Luc/Luc^* and Pgr*^cre/+^* Rosa26*^Luc/+^* mice model of induced endometriosis provides an opportunity to visualize the progesterone receptor (Pgr)-positive cells expressing luciferase (Luc) using bioluminescence imaging (BLI) after administration of D-luciferin. In contrast, the Pgr-negative cells in this model do not express luciferase.

Animal studies were approved by the Institutional Animal Care and Use Committee at Michigan State University and are in compliance with the National Institutes of Health Guide for the Care and Use of Laboratory Animals. All applicable institutional and/or national guidelines for the care and use of animals were followed. IACUC ID PROTO202200313, approved on 9/29/22.

### 2.2. RGD-Cy5.5-MN Synthesis and Characterization

Amine-functionalized dextran-coated iron oxide nanoparticles (MNs) were synthesized and labeled with a near infrared fluorescent Cy5.5 dye according to our previously established procedure [49]. Briefly, 9 g of Dextan-T10 (Pharmacosmos, Holbæk, Denmark) was dissolved in 30 mL of double-distilled water and stirred in a round-bottom flask on ice. Next, 0.65 g of FeCl_3_·6H_2_O (Sigma Aldrich, St. Louis, MO, USA) was added while flushing argon gas into the reaction mixture for an hour. FeCl_2_·4H_2_O (0.4 g, Sigma Aldrich, St. Louis, MO, USA) was added into the mixture, followed by 15 mL of cold 25% NH_4_OH (Acros Organics, Morris Plains, NJ, USA). The temperature was increased to 85 °C for 90 min to induce the formation of a nanoparticulate colloidal mixture, cooled to room temperature, and concentrated to 20 mL using Amicon Ultra centrifugal units (MWCO 30 kDa; Millipore, Dublin, Ireland). The resulting dextran-coated magnetic nanoparticles were cross-linked and aminated through the subsequent addition and stirring of 35 mL of 5 M NaOH (Thermo Fisher Scientific, Fair Lawn, NJ, USA), 14 mL of concentrated (±)-epichlorohydrin (Sigma Aldrich, St. Louis, MO, USA) for 8 h, and 60 mL of concentrated NH_4_OH for the next 36 h. The nanoparticle solution was purified against water using dialysis tubing (MWCO 12–14 kDa, Spectrum Chemical Mfg. Corp., Gardena, CA, USA) and then concentrated by Amicon Ultra centrifugal units in 20 mM citrate buffer (pH ~ 8.0). To conjugate MNs to Cy5.5 fluorescent dye, cynanine5.5 monoreactive NHS ester (Lumiprobe, Hunt Valley, MD, USA) was dissolved in 100 μL of anhydrous dimethyl sulfoxide (Thermo Fisher Scientific, Fair Lawn, NJ, USA) and incubated with MN in 20 mM sodium chloride and sodium citrate buffer (pH ~8.0) overnight. The nanoparticles were purified using a Sephadex PD-10 column (Cytiva, Cardiff, UK) with PBS eluent. c-RGD-conjugated nanoparticles (RGD-Cy5.5-MN) were prepared based on a previously published work [40]. Briefly, to 100 μL of 100 mM suberic acid bis(*N*-hydroxysuccinimide ester) or DSS (Sigma Aldrich, St. Louis, MO, USA) in dimethylformamide (DMF, Thermo Fisher Scientific, Fair Lawn, NJ, USA) was added 5 µL of diisopropylethylamine (Sigma Aldrich, St. Louis, MO, USA). Next, 40 µL of 1 mM c-RGD peptide (sequence: GSSKGGGCRGDC with disulfide bridge 8–12; LifeTein, Hillsborough, NJ, USA) in DMF was added in 5 μL portions to the previous solution and incubated for 1 h. NHS-activated c-RGD peptide was precipitated with m-terbutyl ether (Sigma Aldrich, St. Louis, MO, USA). Cy5.5-MN was added to the precipitate, incubated at 25 °C overnight, and purified from unreacted peptide with a PD-10 column. The size and zeta potential of the particles were determined by dynamic light scattering (Zetasizer Nano ZS, Malvern Instruments, Westborough, MA, USA). The amount of peptide on MN was quantified by a BCA protein assay based on the protocol provided in the kit (Thermo Scientific, Rockford, IL, USA), and iron concentration was determined spectrophotometrically from absorption at 410 nm with iron assay. RGD-Cy5.5-MN was analyzed by transmission electron microscopy (TEM) using the high-resolution JEM-2200FS Field Emission Electron Microscope (JEOL USA Inc., Peabody, MA USA)). The sample was prepared by depositing it onto copper grids and allowing it to air dry. The surface chemistry of the top 50–80 Å was determined with X-ray Photoelectron Spectroscopy (XPS). The measurements were performed using a PHI 5400 ESCA system (Perkin Elmer, Eden Prairie, MN, USA). The base pressure of the instrument was less than 10^-8^ Torr. A 1 cm^2^ sample was mounted onto the sample holder with double-sided copper tape. The X-ray was a monochromatic Al source with a take-off angle of 45 degrees. Regional scans of C1s were acquired at a pass energy of 23.70 eV. Data were fit using the CASA XPS software package (version 2.3.25, Casa Software Ltd., Teignmouth, UK).

### 2.3. Magnetic Resonance Imaging

In vivo MRI was performed in the mouse model of endometriosis 4 to 6 months post endometriosis induction. Mice were divided into two groups: experimental (injected with RGD-Cy5.5-MN; n = 6) and control groups (injected with Cy5.5-MN; n = 3). Imaging was performed on a 7 Tesla Biospec 70/30 USR (Bruker, Billerica, MA, USA) equipped with a 4-channel surface array receive coil (4 × 4 cm) and an 86 mm diameter volume transmit coil. T2*-weighted FLASH sequences with the following parameters: TR/TE = 300/8 ms, 100 µm × 100 µm resolution, 300 µm slice thickness, with fat suppression, field of view of 30 mm × 40 mm, and an average of 10 slices were acquired before and 24 h post probe injection. These nanoparticles have a favorable biodistribution and a blood half-life of 6 h in rodents [50]. Imaging 24 h following probe injection allows for its clearance from the circulation which, in turn, allows for effective visualization of its accumulation in the lesions. Fat-suppressed T2* maps were acquired for quantitative analysis of the probe accumulation with the following parameters: multi-gradient echo sequence, TR/TE = 1500/3.5 ms, 2 averages, 100 µm × 100 µm resolution, 300 µm slice thickness, flip angle 50°, 10 echo images with minimum echo time of 2.62 ms and 4.8 ms spacing, 30 mm × 40 mm field of view, and respiratory gating. Temperature (~35 °C) and breathing were continually observed and sustained during the course of the experiment (SAII Small Animal Instruments, Inc., Stony Brook, NY, USA). First, pre-contrast images were acquired followed by intravenous injection of RGD-Cy5.5-MN or Cy5.5-MN (10 mg Fe/kg). Mice were imaged again 24 h post-injection with the same parameters. Image analysis was conducted using Paravision 360 v3.2 software (Bruker BioSpin, Ettlingen, Germany). The ROIs of the muscle tissue served as controls within each animal group. T2* before injection minus T2* after injection (delta T2*, ms) were used to evaluate the accumulation of probes in the lesions.

### 2.4. Ex Vivo Bioluminescence Optical Imaging

To verify the location of luciferase-expressing lesions detected with MR imaging, immediately after the last MR imaging session, mice were injected intraperitoneally with IVISbrite D-Luciferin potassium salt in PBS (100 µL of 300 mg/mL, Perkin Elmer, Boston, MA, USA). Ten minutes after injection, mice were euthanized and collected tissues were subjected to bioluminescence imaging (BLI) using an IVIS Spectrum imaging system (Perkin Elmer, Hopkinton, MA, USA). BLI was performed with the following settings: exposure time, 30 s; f number, 8; binning factor, 2. All images were processed using the Living Image Software (version 4.5.2, Perkin Elmer, Hopkinton, MA, USA).

### 2.5. Ex Vivo Epifluorescence Optical Imaging

To evaluate the biodistribution of nanoparticles, after acquisition of ex vivo bioluminescence images we performed epifluorescence optical imaging on collected tissues of mice injected with either RGD-Cy5.5-MN or Cy5.5-MN. The acquisition conditions are summarized as follows: exposure time, 30 s; binning factor, 2; excitation filter range, 675 nm; emission filter range, 720 nm; f number, 8. All images were analyzed using the Living Image Software (version 4.5.2, Perkin Elmer). Imaging data were normalized, and quantified signal was expressed as radiant efficiency ([p/s/cm^2^/sr]/[µW/cm^2^]).

### 2.6. ICP-OES Analysis

To quantitate the iron content in the collected lesion and muscle tissues, samples were dried, weighed, and digested with 69% nitric acid (TraceSELECT™, Fluka, St. Louis, MO, USA) at room temperature. The iron content in each sample was determined through inductively coupled plasma optical emission spectrometry (ICP-OES) utilizing a 710-ES spectrometer (Varian, Palo Alto, CA, USA). Blank nitric acid and calibration samples containing a predetermined iron concentration were prepared using TraceCERT^®^ Iron (Fe) Standard for ICP (1000 mg/L Fe in nitric acid, Sigma-Aldrich, St. Louis, MO, USA) and analyzed alongside the test tissue samples. Measurements were carried out in triplicate, and the resulting data were normalized to the dry tissue weight and presented as mean ± SD.

### 2.7. Histology and Immunostaining

To analyze endometriotic lesions, dissected tissues were embedded in optimal cutting temperature compound (OCT, Sakura Finetek, Torrance, CA, USA) and rapidly cryopreserved using liquid nitrogen. The tissues preserved in OCT were cut into 10 μm sections, fixed with 10% neutral buffered formalin (Fisherbrand, Pittsburg, PA, USA), blocked in a 5% normal goat serum in phosphate-buffered saline, and stained for CD34—an endothelial marker—with rat monoclonal anti-mouse CD34 antibody (1:100 dilution; Abcam, Cambridge, MA, USA), followed by Alexa Fluor 594-conjugated goat anti-rat (1:400 dilution; Invitrogen, Carlsbad, CA, USA), and mounted with a DAPI (4,6-diamidino-2-phenylindole) containing mounting medium (Vectashield, Vector Laboratories, Burlingame, CA, USA) for nuclear staining. To compare nanoparticle accumulation, tissue sections were analyzed by fluorescence microscopy using a Nikon Eclipse 50i fluorescence microscope (Nikon, Tokyo, Japan) equipped with the Cy5.5 filter sets (Chroma Technology Corporation, Bellows Falls, VT, USA). Images were acquired using a charge-coupled device camera with NIRF sensitivity (SPOT 7.4 Slider RTKE; Diagnostic Instruments, Sterling Heights, MI, USA). The images were analyzed using SPOT 5.2 Advance version software (Diagnostic Instruments, Sterling Heights, MI, USA). The ratio of the red to the blue signal in the images reflecting RGD-Cy5.5-MN and Cy5.5-MN accumulation in the experimental and control lesions, respectively, was measured and normalized using Image J software (version 1.54e 4, U.S. National Institutes of Health, Bethesda, MD, USA), with the red signal depicting Cy5.5 conjugated nanoparticles and the blue signal depicting cell nuclei.

### 2.8. Complete Blood Count, Blood Chemistry Analyses, and Histopathology

A comprehensive study of blood parameters, including a complete blood count (CBC) and blood chemistry profile, was conducted using two groups of female mice with the same genetic background as animals used in the imaging studies. The first group received injections of RGD-Cy5.5-MN at a dosage of 10 mg/kg, while the second group served as a non-injected control. Twenty-four hours after injection, animals were anesthetized, and their blood was collected via cardiac puncture. Following euthanasia, major organs including the liver, spleen, lungs, heart, and kidneys were excised, fixed in a 10% formalin solution, processed, cut, and stained with H&E stain. All analyses, including the blood measurement panel and histopathological interpretation, were performed by the Veterinary Diagnostic Laboratory, College of Veterinary Medicine (Michigan State University).

### 2.9. Statistical Analysis

All data are represented as mean ± SD. Statistical analysis was performed using a two-tailed Student’s *t*-test. *p* < 0.05 was considered statistically significant.

## 3. Results

### 3.1. Synthesis and Characterization of the Nanoparticle Probes

Iron-oxide-based dextran-coated magnetic nanoparticles (MN) were synthesized and conjugated to Cy5.5 near infrared fluorescent dye resulting in 8 dye molecules per nanoparticle determined by spectrophotometry. The number of cRGD peptides per nanoparticle after conjugation to Cy5.5-MN was 12 as measured by BCA assay. The size and zeta potential of the probes were 25 ± 13 nm and 15 mv, respectively, for Cy5.5-MN and 33 ± 8 nm and 19 mv, respectively, for RGD-Cy5.5-MN. RGD-Cy5.5-MN and Cy5.5-MN characterization is summarized in Table S1. Figure S1 features a representative transmission electron microscopy (TEM) image, depicting RGD-Cy5.5-MN nanoparticles, with a measured core size of 5.8 ± 0.4 nm. The increase in the peak area percentage of N-C=O and C=O in the XPS high-resolution C1s spectrum of RGD-Cy5.5-MN (4.7%) compared to Cy5.5-MN (2.7%) can be linked to the attachment of the RGD peptide to the surface of iron oxide nanoparticles, consequently leading to an increased number of amide bonds (Figure S2).

### 3.2. Magnetic Resonance Imaging of Endometriotic Lesions

**Figure 1:**
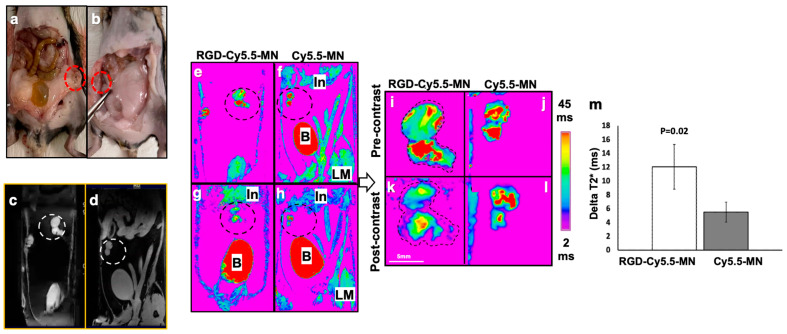
Representative photographs and T2* maps of the lesions in a mouse model of endometriosis. (**a**,**b**) Representative ex vivo photographs of endometriotic lesions shown within the red dotted circles. (**c**,**d**) Representative pre-contrast T2* maps (black and white color-coded) with endometriotic lesions shown within white dotted circles. (**e**) Pre-contrast and (**g**) post-contrast T2* maps of animals injected with RGD-Cy5.5-MN. (**f**) Pre-contrast and (**h**) post-contrast T2* maps of animals injected with Cy5.5-MN. (**i**) Magnified T2* maps of the lesions before and (**k**) after RGD-Cy5.5-MN administration. (**j**) Magnified T2* maps of the lesions captured before and (**l**) after Cy5.5-MN administration. Dotted area shows borders of the lesions. (**m**) Quantitative data (delta T2*) from the images (T2* maps) in (**i**–**l**). B—bladder; In—intestine; LM—leg muscle. Endometriotic lesions are shown within blue dotted circles in (**e**–**i**,**k**).

In vivo MR imaging was performed on animals with endometriotic lesions before and 24 h after the injection of the probes. Representative ex vivo photographs of endometriotic lesions are shown within the red dotted circles (Figure 1a,b and Figure S3) and representative pre-contrast MR T2* maps (Figure 1c,d) in black and white are shown for better visualization of the relative location of lesions in the mouse peritoneum. Figure 1e,g display representative pre- and post-contrast color-coded T2* maps, respectively, of animals injected with RGD-Cy5.5-MN, while Figure 1f,h show representative pre- and post-contrast T2* maps, respectively, of animals administered with the control probe (Cy5.5-MN). For enhanced visualization, enlarged representative color-coded T2* maps of endometriotic lesions are provided for both the experimental group (pre-contrast: Figure 1i, post-contrast: Figure 1k) and the control group (pre-contrast: Figure 1j, post-contrast: Figure 1l). Pre- and post-T2*-weighted images showing the areas of signal loss (hypointensity) induced by RGD-Cy5.5-MN accumulation are shown in Figure S4. These images qualitatively depict a more pronounced decrease in the T2* relaxation time of the group injected with RGD-Cy5.5-MN compared to the control group 24 h after injection. This indicates a higher accumulation of RGD-Cy5.5-MN in the lesions compared to control nanoparticles. Quantitative analysis of the T2* map validated the qualitative data, showing a significantly larger change in T2* relaxation times in the lesions of animals injected with RGD-Cy5.5-MN compared to the lesions of the control group (*p* < 0.02; Figure 1m). There was no significant change in the T2* relaxation times of the muscle tissue before and 24 h post-injection in either the experimental or control group (0.65 ± 0.6 ms vs. 1.5 ± 2.6 ms respectively, *p* > 0.05).

### 3.3. RGD-Cy5.5-MN Biodistribution

**Figure 2:**
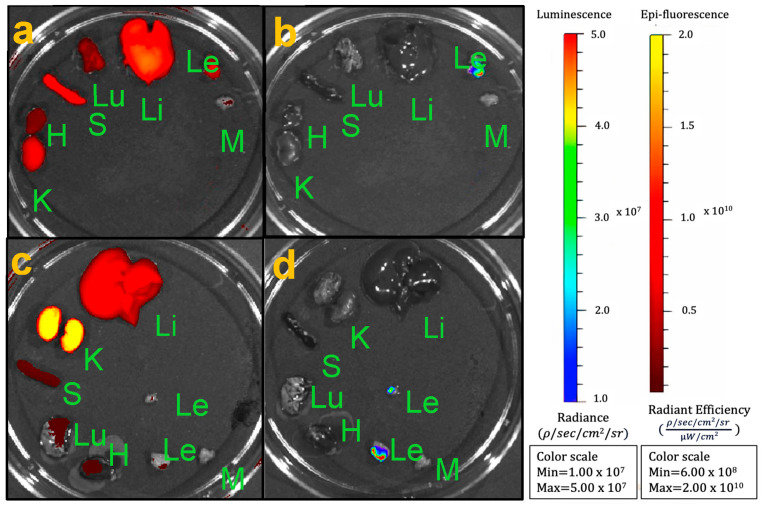
Ex vivo biodistribution studies. (**a**,**c**) Fluorescence imaging of the biodistribution assessment of (**a**) experimental RGD-Cy5.5-MN and (**c**) control Cy5.5-MN probes; (**b**,**d**) Bioluminescence imaging showing the signal coming only from luciferase-expressing lesions in experimental and control groups. K—kidney; H—heart, S—spleen, Lu—lungs; Li—liver; Le—lesion; M—muscle.

To demonstrate the accumulation of RGD-Cy5.5-MN and Cy5.5-MN in different organs, ex vivo biodistribution studies were performed for both experimental and control groups of animals using fluorescence optical imaging (Figure 2a,c). Bioluminescence optical imaging was performed to validate the presence of Cy5.5-labeled nanoparticles in luciferase-expressing endometriotic lesions in both the experimental and control groups (Figure 2b,d).

**Figure 3:**
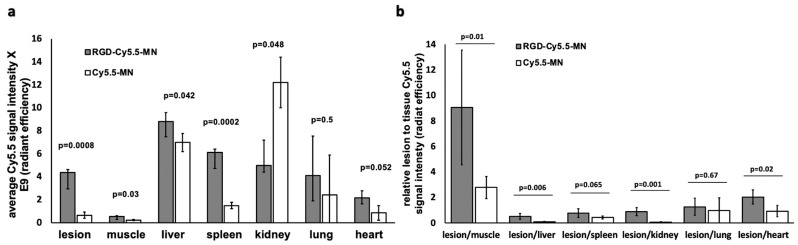
(**a**) Quantification of ex vivo biodistribution shows significantly higher lesion accumulation of RGD-Cy5.5-MN compared to the control group. (**b**) Cy5.5 signal of the lesion normalized to the signal from other organs shows a significantly higher ratio when normalized to muscle, kidney, and heart.

Figure 3a shows the Cy5.5 signal from both probes accumulated in different organs normalized to the organ surface area. Quantitative analysis demonstrated that accumulation of RGD-Cy5.5-MN in the lesions was significantly higher compared to Cy5.5-MN accumulation (*p* = 0.0008). Furthermore, this accumulation was significantly higher than accumulation in the muscle tissues in both experimental and control groups (*p* = 0.001). Comparing biodistribution to other organs in the experimental and control groups, we observed a significant difference in accumulation of the probes between internal organs such as the liver and spleen. This is not unexpected and can be due to expression of alpha(v)beta3 on the cell surface of activated tissue macrophages of the reticuloendothelial system (RES) described earlier [51,52]. The changes in the RES uptake have been observed in several previous studies investigating RGD-conjugated nanocarriers [51,53]. Despite the increased accumulation of RGD-conjugated nanoparticles in non-targeted tissues, our results show higher lesion-to-organ Cy5.5 signals in mice injected with the targeted probe compared to control nanoparticles when the lesion signal was normalized to the signal from other tissues—including muscle, liver, kidney, and heart—demonstrating considerable potential of RGD-Cy5.5-MN for endometriotic lesion targeting (Figure 3b).

**Figure 4:**
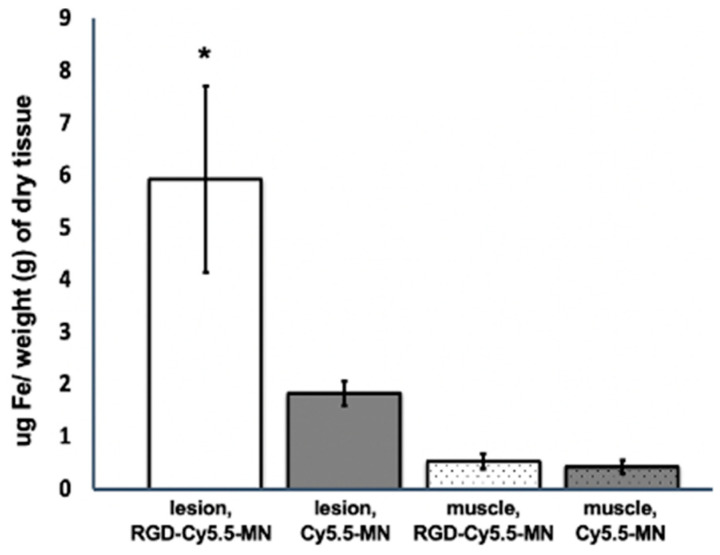
ICP-OES analysis of the average iron content in endometriotic lesions and muscle tissues after intravenous administration of experimental (RGD-Cy5.5-MN) or control (Cy5.5-MN) probe. The iron content within the tissues was normalized to the dry weight of the tissues. Results are presented as means ± SD. Note that the lesions in animals injected with the experimental probe showed a higher iron content compared to that in animals injected with the control probe. The asterisk signifies a statistical significance (*p* < 0.01) in the comparison of iron content between lesions in the experimental and control groups.

To corroborate the higher accumulation of RGD-Cy5.5-MN in lesions of experimental mice compared to lesions from mice injected with Cy5.5-MN signified by a T2* relaxivity drop, tissue samples containing endometriotic lesions and muscle tissues from both animal groups were analyzed for the presence of iron by ICP-OES (Figure 4). The results indicated that the iron content in endometriotic lesions of experimental group (5.9 ± 1.7 µg Fe/g dry tissue) was significantly higher compared to both skeletal muscle of the same group (0.52 ± 0.14 µg Fe/g dry tissue) and lesions of the control group (1.8 ± 0.22 µg Fe/g dry tissue). There was no significant difference between iron content in control muscle tissues from both experimental and control groups.

### 3.4. Ex Vivo Analysis of RGD-Cy5.5-MN Accumulation and CD34 Expression

**Figure 5:**
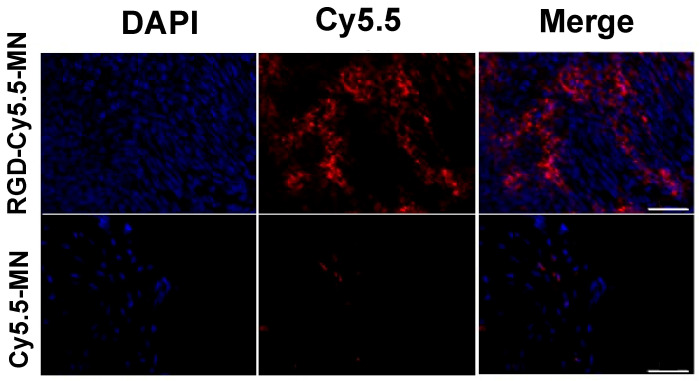
Fluorescence microscopy demonstrates a relatively higher accumulation of RGD-Cy5.5-MN (**top** row) in endometriotic lesions compared to Cy5.5-MN (**bottom** row). Blue—DAPI nuclear stain; red—Cy5.5. Bar = 50 µm.

To further confirm our in vivo MRI results, lesion sections from both experimental and control groups of mice were analyzed with fluorescence microscopy. Accumulation of RGD-Cy5.5-MN in lesions from the experimental group

(Figure 5, top row), was visually higher compared to accumulation of Cy5.5-MN in the lesions of control group (Figure 5, bottom row). Quantitatively, the experimental group exhibited an average ratio of 0.54 ± 0.18 of RGD-Cy5.5-M, N accumulation which was significantly higher compared to the accumulation of Cy5.5-MN in the lesions of control group (0.13 ± 0.048, *p* = 0.01). These findings are in line with the results obtained by MR imaging.

**Figure 6:**
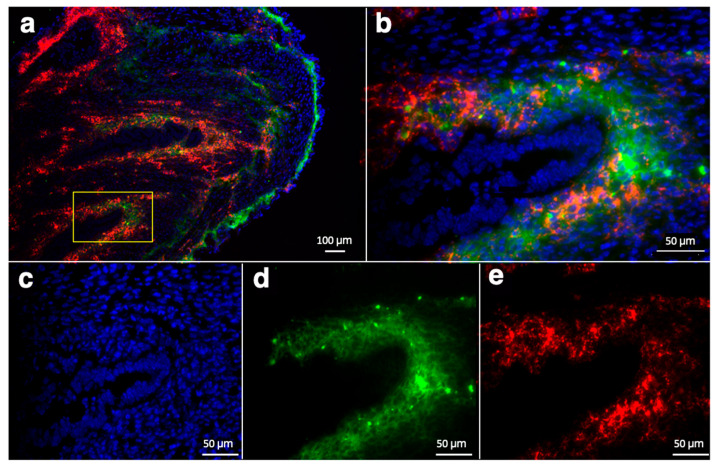
Immunofluorescence staining of endometriotic lesion sections from animals injected with RGD-Cy5.5-MN shown with (**a**) low; bar = 100 μm and (**b**) high; bar = 50 μm magnification. Note that (**b**) shows the area within the yellow rectangle in (**a**). Cell nucleus DAPI staining (**c**), CD34 marker (**d**, green) and Cy5.5 (**e**, red) are shown separately in the bottom row.

Additionally, staining for the endothelial marker CD34 [54,55] clearly showed colocalization with the Cy5.5 signal suggesting that accumulation of RGD-Cy5.5-MN was specific to overexpression of alpha(v)beta3 integrins [56] and is not solely due to passive targeting (Figure 6a,b).

### 3.5. Biosafety Assessment

With the outlook to clinical translation of our approach, we performed biosafety assessment of the probe. There were no statistically significant differences detected in the measured analytes for both the complete blood count (CBC) and blood chemistry profile analyses in the group injected with RGD-Cy5.5-MN compared to the non-injected controls. The summary of the analytes’ values is presented in Tables S2 and S3. Even though we found that sodium, albumin, and total protein levels showed significant differences between the experimental and control groups, their values fell within the normal range according to the previously published data [57]. As expected, the iron in the experimental group (injected with RGD-Cy5.5-MN) was elevated to 200.6± 14.9 µg/dL 24 h post-injection. Based on H&E staining, there were no significant pathological differences in the major organs, including the heart, liver, spleen, lungs, and kidneys of both groups (Figure S5).

## 4. Discussion

Endometriosis is a heterogeneous disease which, as of today, has no clinically approved biomarkers for noninvasive diagnosis [58]. A clinical trial (NCT03376451) designed to analytically validate a cluster of specific biomarkers for endometriosis diagnosis and disease recurrence has yet to publish their results. Although different noninvasive clinical imaging modalities are being used to diagnose endometriosis, they cannot detect all types and sizes of lesions [59]. A recent review on imaging endometriosis emphasized poor methodological quality of the studies and the absence of imaging methods capable of detecting endometriosis with as much accuracy as surgical evaluation [15]. Targeted delivery of contrast agents to endometriotic lesions could potentially resolve this problem and assist in detection of lesions using various imaging modalities including MRI. One approach for targeted delivery of contrast agents to the lesions could involve developing nanocarriers with ligands targeting specific and/or overexpressed biomarkers in the lesions [26,60,61]. Endometriosis heavily relies on adhesions and angiogenic factors for implantation and survival of the lesions [29], therefore, angiogenesis can play a role of a surrogate imaging biomarker for this disease. Several studies have already shown the presence of alpha(v)beta3 integrin in endometriotic lesions [62,63,64], which can be targeted with the RGD peptides [38,65]. In this study, we hypothesized that c-RGD conjugation can enhance accumulation of iron oxide nanoparticles in the lesions by targeting alpha(v)beta3 integrin leading to an improvement in lesion detection by MRI. For this purpose, cyclic RGD peptide was conjugated to optically labeled MR biocompatible iron oxide nanoparticles and tested for imaging endometriotic lesions in a mouse model of endometriosis. In our studies, we used a model with established lesions and developed vasculature. The reason for that is that one of the main symptoms of endometriosis is pain [4], which is commonly misinterpreted by patients as menstrual cramps. Many patients experience this pain for years after the disease initiation before seeking medical advice, and eventually present the physician with older, developed lesions. This disease presentation demonstrates the necessity for a probe that can identify established lesions with considerable amount of angiogenesis.

MR imaging and comparison of the average delta T2* values from experimental and control groups of animals supports our hypothesis showing a significantly larger reduction in the lesions of the animals injected with RGD-Cy5.5-MN. To further corroborate our MRI data, we performed ex vivo biodistribution. Comparing the two groups, a significant difference was observed in several other organs including spleen, liver, and muscle, which can be attributed to the variability of expression of integrins on RES cells [51,53]. Previous studies also showed that the number of RGD molecules on the nanocarrier has an effect on its binding capacity to different cells, organs and its half-life in blood [40,66]. Biodistribution data also showed significantly higher numbers when Cy5.5 signal from nanoparticles accumulated in lesions from the group injected with RGD-Cy5.5-MN was normalized to the signal from the tissues and organs. ICP-OES findings also revealed significantly elevated iron content in the lesions from the experimental group compared to the control group which supports data acquired from MRI and ex vivo fluorescence imaging. Histological analysis of the lesions from experimental and control groups showed a higher accumulation of RGD-Cy5.5-MN with the majority of the probe accumulating in the areas expressing the angiogenesis marker, CD34. It has been previously shown that CD34^+^ cells express alpha(v)beta3 integrin [67], which was our target of interest. It is noteworthy that there are various studies quantifying expression of alpha(v)beta3 integrin in both the eutopic and ectopic endometrium of human or animal models of endometriosis [68,69]. However, since not all the data are in complete agreement, a more comprehensive study on the expression of integrins in this disease is needed [62,63,64]. Currently, an ongoing clinical trial (NCT05623332) which is scheduled to complete in 2024 is investigating the presence of integrins in endometriotic tissue. This is expected to expand our understanding of endometriosis and could be used for developing a non-invasive imaging method in the future.

Established toxicological and pharmacological evaluations of various iron oxide-based contrast agents demonstrated their favorable safety profile for human use, considering the inherent presence of iron in human tissues. Protective coating for the nanoparticles used in our study (dextran) showed safe characteristics as well [70,71]. Furthermore, an RGD peptide, as a tumor-targeting moiety, has shown a positive safety profile as assessed by liver and kidney function tests as well as by histopathology in previous studies [72,73]. Our in vivo toxicity assessment was in line with these findings and showed no pathology in CBC or blood chemistry panels, as well as in the pathological examination of major organs (including the liver, heart, lungs, spleen, and kidneys) when comparing the group of mice injected with RGD-Cy5.5-MN to the group of untreated mice. These findings serve as a solid basis for future comprehensive toxicology studies, which will include the RGD conjugated nanoparticles without Cy5.5 according to the FDA requirements. Even though the presence of pain is one of the distinguishing symptoms that sets endometriosis apart from cancers, it fluctuates with the menstrual cycle [74]. Since similar RGD-conjugated imaging probes have been previously used in cancer imaging [38] we plan to conduct studies comparing the enhancement patterns of endometriotic lesions and cancerous tumors, aiming to improve differentiation between these two pathologies.

In summary, our data demonstrates that cyclic RGD-conjugated optical/MRI-sensitive iron oxide nanoparticles can be used to efficiently target ectopic endometriotic lesions. In this study, we chose to use a well-established suture model of endometriosis commonly utilized in preclinical studies [75]. The main rationale for employing this model is that it provides the exact location of the lesions, enabling their identification in our proof-of-concept imaging studies. Based on these studies, we plan to validate our findings in the peritoneal injection mouse model [48] in the future followed by a non-human primate model [76,77] in which the locations of the lesions are random and closely mirror a clinical scenario [78].

## Data Availability

Data are contained within the article and Supplementary Materials.

## Supplementary Materials

The following supporting information can be downloaded at: https://www.mdpi.com/article/10.3390/nano14030319/s1.
