# Supplementary material for: Imaging of Endometriotic Lesions Using cRGD-MN Probe in a Mouse Model of Endometriosis
Source: Nanomaterials (Basel). 2024 Feb 5;14(3):319. doi: 10.3390/nano14030319 (PMC10856945; doi:10.3390/nano14030319)
Supplement: Supplementary file 1 [file nanomaterials-14-00319-s001.zip › nanomaterials-2814469-supplementary.pdf]

Supplementary Materials

Imaging of Endometriotic Lesions Using cRGD-MN Probe in a Mouse Model of Endometriosis

Nazanin Talebloo [1,2], M. Ariadna Ochoa Bernal [3,4], Elizabeth Kenyon [1,5], Christiane L. Mallett [5,6], Sujan Kumar Mondal [1,5], Asgerally Fazleabas [3] and Anna Moore [1,5,*]

1 Precision Health Program, Michigan State University, 766 Service Road, East Lansing, MI 48824, USA; talebloo@msu.edu (N.T.); kenyonel@msu.edu (E.K.); mondalsu@msu.edu (S.K.M.)
2 Department of Chemistry, College of Natural Sciences, Michigan State University, 578 S Shaw Lane, East Lansing, MI 48824, USA
3 Department of Obstetrics, Gynecology & Reproductive Biology, Michigan State University, 400 Monroe Avenue NW, Grand Rapids, MI 49503, USA; ochoaber@msu.edu (M.A.O.B.); fazleaba@msu.edu (A.F.)
4 Department of Animal Science, Michigan State University, 474 S Shaw Ln #1290, East Lansing, MI 48824, USA
5 Department of Radiology, College of Human Medicine, Michigan State University, 766 Service Road, East Lansing, MI 48824, USA; cmallett@msu.edu
6 Institute for Quantitative Health Science and Engineering, Michigan State University, 775 Woodlot Drive, East Lansing, MI 48824, USA
* Correspondence: moorea57@msu.edu

Figure S1

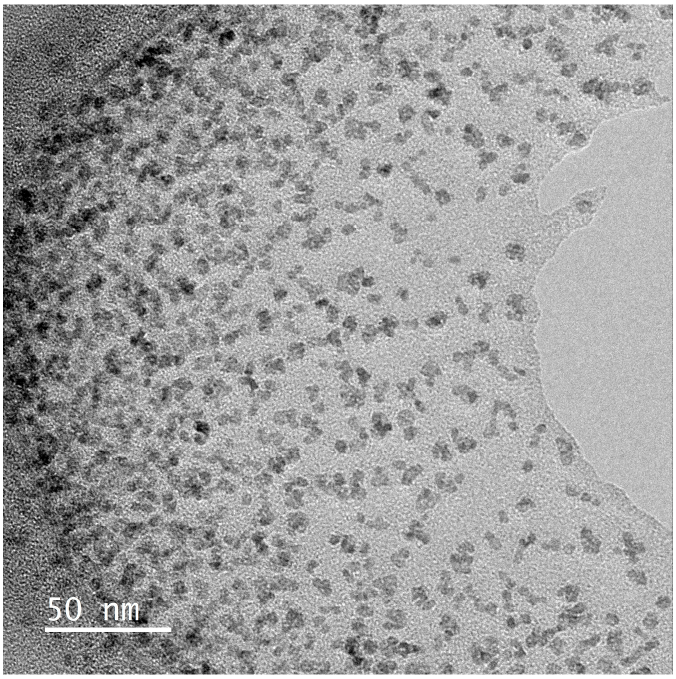

Figure S1. Representative transmission electron microscopy (TEM) image of RGD-Cy5.5-MN captured on high-resolution JEM-2200FS Field Emission Electron Microscope (JEOL). For imaging, the sample was prepared by placing RGD-Cy5.5-MN into the copper grids and air dried. The size of RGD-Cy5.5-MN core was measured 5.8 ± 0.4 nm. Scale bar = 50 nm.

Figure S2

RGD-Cy5.5-MN C1s spectra **Cy5.5-MN C1s spectra**

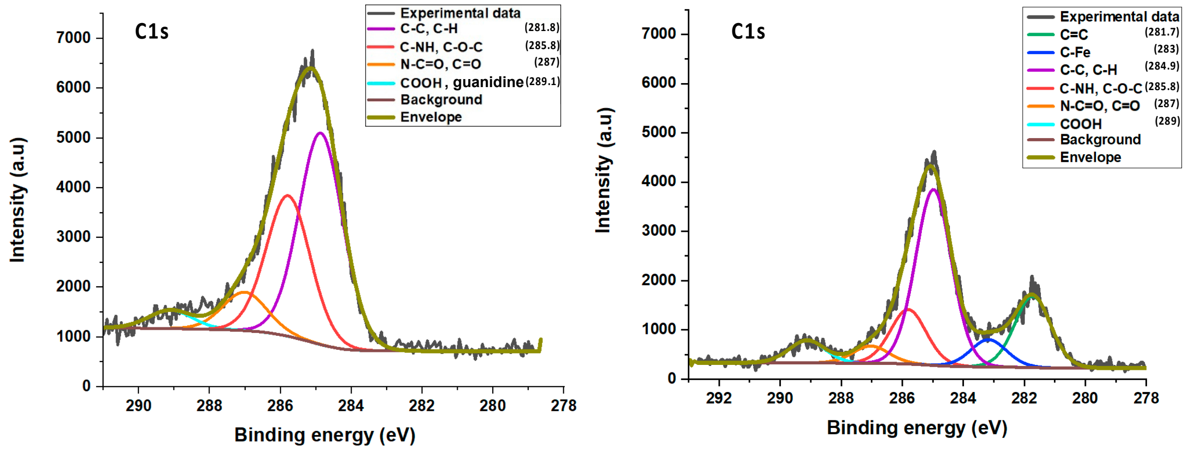

Figure S2. a) The high-resolution C1s spectrum of RGD-Cy5.5-MN, and b) C1s spectra of Cy5.5-MN. An increase in 287 ev peak can be attributed to an increase in amide bond due to RGD peptide conjugation to the iron oxide nanoparticles. C-Fe cannot be detected in RGD-Cy5.5-MN which can be due to the coated surface of particles after conjugation with the peptide.

Figure S3

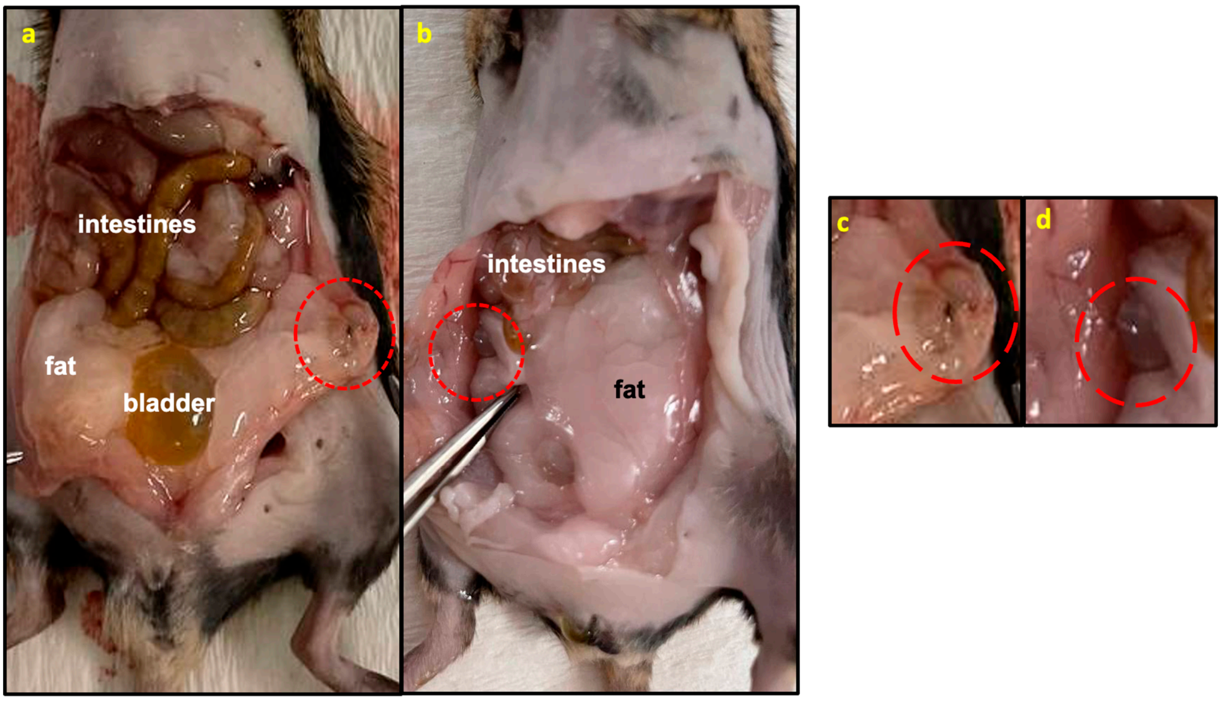

Figure S3. a) and b) are representative ex vivo photographs of endometriotic lesions shown within red dotted circles. Various organs are shown in these images. c) and d) are enlarged lesions for enhanced visualization.

Figure S4

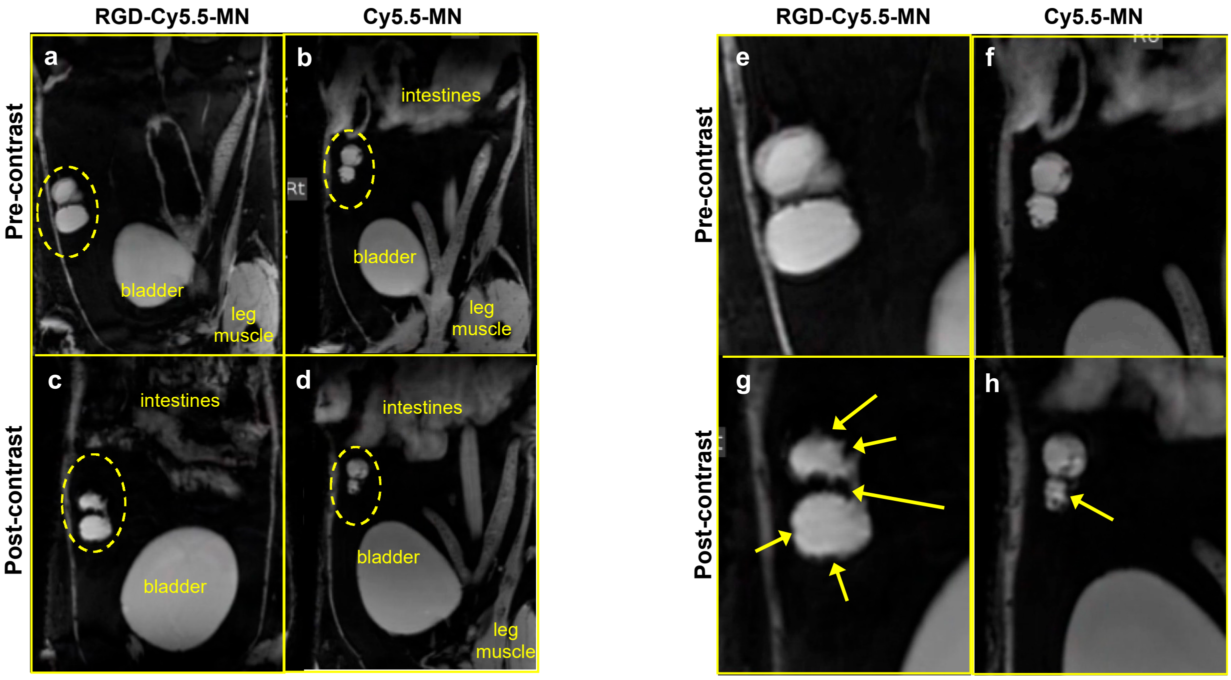

Figure S4. a) Pre-contrast and c) post-contrast T2*-weighted MR images of the animals injected with RGD-Cy5.5-MN. b) Pre-contrast and d) post-contrast images of animals injected with Cy5.5-MN. Lesions are shown within yellow dotted circles. e) Enlarged images of lesions captured e) before and g) after RGD-Cy5.5-MN administration. Enlarged images of lesions captured f) before and h) after Cy5.5-MN administration. The darkening effect is shown with yellow arrows.

Figure S5

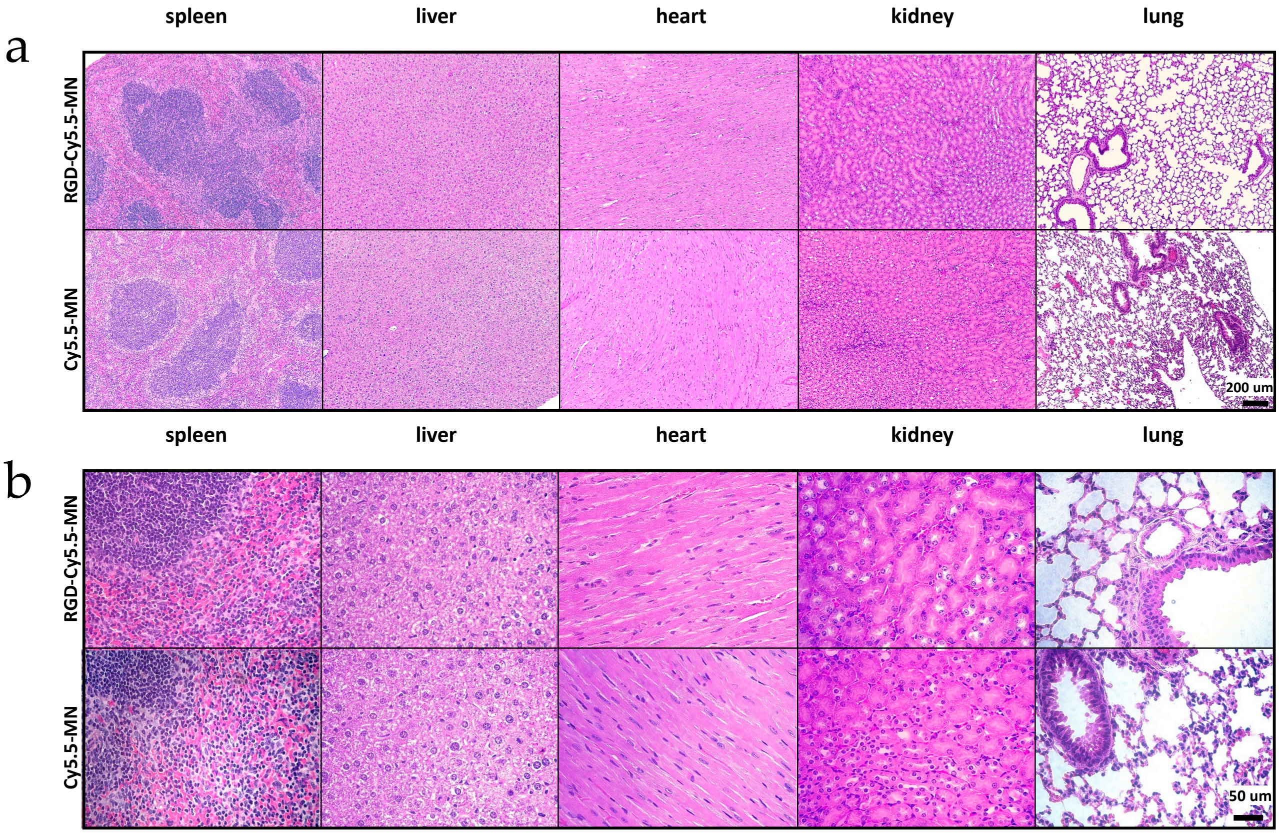

Figure S5. H&E staining of mice major organs (spleen, liver, heart, kidney, lung) from mice injected with RGD-Cy5.5-MN (top row) or mice that did not undergo any injections (bottom row) in **a)** low magnification, magnification bar: 200 um and **b)** higher magnification, magnification bar: 50 um.

Table S1. Summary of Cy5.5-MN and RGD-Cy5.5-MN characterization

Characterization	Size	polydispersity index (PDI)	Number of amines per particle	Zeta potential	Number of Cy5.5 dyes per particle	Number of peptides per particle
Cy5.5-MN	25 ± 13nm	0.25	105 (before Cy5.5 conjugation)	+15 mv	8	_
RGD-Cy5.5-MN	33 ± 8nm	0.14	_	+19 mv	8	12

Table S2. Complete blood count (CBC)

Analyte	Difference between experimental & control groups (NS=not significant)
RBC	NS
Hgb	NS
Hct (Hematocrit)	NS
MCV (Mean Corpuscular Volume)	NS
MCH (Mean Corpuscular Hemoglobin)	NS
MCHC (Mean Corpuscular Hemoglobin Concentration)	NS
CHCM (Cell Hemoglobin Concentration Mean)	NS
RDW (Red Cell Distribution Width)	NS
Total Retic Pct (Total Reticulocyte Percentage)	NS
Total Retic # (Total Reticulocyte Count)	NS
CHr (Hemoglobin Content of Reticulocytes)	NS
Platelet	NS
MPV (Mean Platelet Volume)	NS
WBC	NS
Seg Neut # Manual	NS
Lymphocyte # Manual	NS
Monocyte # Manual	NS
Seg Neut Pct Manual	NS
Lymphocyte Pct Manual	NS
Monocyte Pct Manual	NS

Table S3. Blood Chemistry.

Analyte	Difference between experimental & control groups (NS: not significant)
Urea Nitrogen (mg/dL)	NS
Creatinine (Jaffe) (mg/dL)	NS
Sodium (mmol/L)	**P=0048** All experimental and control data are within the previously published data of female C57/BL6 mice. (Charles River Health Profiles for C57BL/6)
Potassium (mmol/L)	NS
Chloride (mmol/L)	NS
TCO2 (mmol/L)	NS
Na/K Ratio	NS
Anion Gap (mmol/L)	NS
Osmolarity Calc (mmol/L)	NS
Calcium (mg/dL)	NS
Phosphorus (mg/dL)	NS
Magnesium (mg/dL)	NS
Total Protein (g/dL)	**P=0.006** All experimental and control data are within the previously published data for female C57/BL6 mice [57].
Albumin (g/dL)	**P=0.0056** All experimental and control data are within published data for female C57/BL6 mice (Charles River Health Profiles for C57BL6)
Globulin Calc (g/dL)	NS
Glucose (Hexokinase) (mg/dL)	NS
Amylase (U/L)	NS
Total Bili (mg/dL)	NS
Indirect Bili (mg/dL)	NS
ALP (U/L)	NS
ALT (U/L)	NS
AST (U/L)	NS
CK (U/L)	NS
Chol (mg/dL)	NS
